# A Study on the Patterns of Psychiatric Referrals in a Tertiary Care Hospital in the North-Eastern Part of India

**Published:** 2020-08

**Authors:** Suresh Chakravarty, Siddhartha Nandi, Samrat Singh Bhandari, Shyamanta Das

**Affiliations:** 1 Department of Psychiatry, Gauhati Medical College Hospital, Guwahati, Assam, India; 2 Department of Psychiatry, Gauhati Medical College Hospital, Guwahati, Assam, India; 3 Department of Psychiatry, Sikkim Manipal Institute of Medical Sciences, Gangtok, Sikkim, India; 4 Department of Psychiatry, Gauhati Medical College Hospital, Guwahati, Assam, India

**Keywords:** Consultation-Liaison Psychiatry, General Hospital, Deliberate Self-Harm, Use of Alcohol

## Abstract

**BACKGROUND:**

Consultation-liaison psychiatry holds a special place, especially in general hospital setting. We wanted to study the socio-demographic variables, reasons for referral, and clinical correlates of psychiatric referrals from different wards in a tertiary care hospital.

**METHODS:**

This was a retrospective chart-review study carried out in the Gauhati Medical College Hospital, Guwahati, for a period of one month (May 2019). Demographic data of the participants was collected. Their primary medical / surgical diagnoses were noted along with the department where they were admitted. The reason for psychiatric consultation as well as the psychiatric diagnoses were analysed by descriptive statistics with the use of GraphPad InStat.

**RESULTS:**

Most of the sample was men (men: women: 106:72), mostly between 11 to 30 years (44.38%) and Hindus (Hindu: Muslim: 140:38). Majority of the consultations was from the Department of Medicine (47.19%), followed by Orthopaedics (15.17%) and Surgery (14.61%). Among the surgical/medical illnesses, most of them had fractures (ten), closely followed by neurological conditions like cerebrovascular accident (five) and head injury (four). Deliberate self-harm was the most common reason for psychiatric liaison (56), that constituted a high number with poisoning (45) as well as hanging (six) and cut neck (five). Psychiatric consultation was asked for use of alcohol in a substantial number of patients (45).

**CONCLUSIONS:**

Addressing the psychiatric comorbidity while continuing treatment for the surgical/medical illnesses gives a holistic approach towards our patients that can influence not only the course but also the quality of life of them.

## BACKGROUND

The department of psychiatry plays a multi-dimensional role in a general hospital. The department provides outdoor and indoor services to the psychiatric patients. Along with that it also attends to the psychiatric disorders of the patients primarily having medical/surgical illness. The later role is provided through the consultation-liaison psychiatric services.^[1,2]^ Today’s digital era has made the world a global village. Literally, with the click of the mouse we can appraise ourselves about the happenings around the globe. This is pertinent too to keep ourselves vibrant in the rapidly changing scenario. At the same time, there is the need to remain aware of the fact that cultural factors specific to a geography do have influences. Therefore, while applying a globally acceptable approach to a particular geography we need to take into consideration the relevant cultural issues. Equally, once such an approach becomes successful it needs to be placed in front of a wider audience for better acceptability and replicability. This two-way communication between global and local needs led a school of thought to coin the term ‘glocalisation’ to represent the picture of globalisation.^[3]^ Literature on consultation-liaison psychiatry is scarce from northeast India.^[4,5]^ Keeping the concept of glocalisation in mind, we wanted to present data on the subject from this region to a wider audience along with literature search.

### Objectives

1. To study the demographic profile of patients admitted in different departments of a general hospital who are referred for psychiatric consultation.
2. To study which are the departments that sought psychiatric consultations.
3. To study the reasons for such psychiatric consultations.
4. To study the primary medical/surgical illnesses for which the patients are admitted in the parent departments.

## METHODS

This is a retrospective chart-review study. The study was carried out in the Gauhati Medical College Hospital (GMCH) located in Guwahati, the capital of Assam, one of the eight north-eastern states of India for a period of one month, i.e. May 2019. Patients admitted in different departments of GMCH for whom psychiatric consultation was asked for during the study period were the participants. Demographic data of the participants were collected. Their primary medical/surgical diagnoses were noted along with the department where they were admitted. The reason for psychiatric consultation as well as the psychiatric diagnoses were analysed.

### Data Sources / Measurement

Data was collected from available charts in the Department of Psychiatiy, GMCH and entered into a proforma prepared for the study after decoding the identifying information of the participants.

### Bias

In retrospective chart-review studies, there remained the possibility of missing data.

### Study Size

All psychiatric consultations during the study period were included and the sample size was 178.

### Statistical Methods

Statistical analysis was done by descriptive methods in the form of mean, standard deviation, frequency, and percentage.

### Ethical Clearance and Informed Consent

The study being of retrospective chart-review design, it was exempt from ethical clearance and informed consent. Administrative permission was taken from the Head of the Department, Department of Psychiatry, GMCH.

## RESULTS

**Figure 1.**
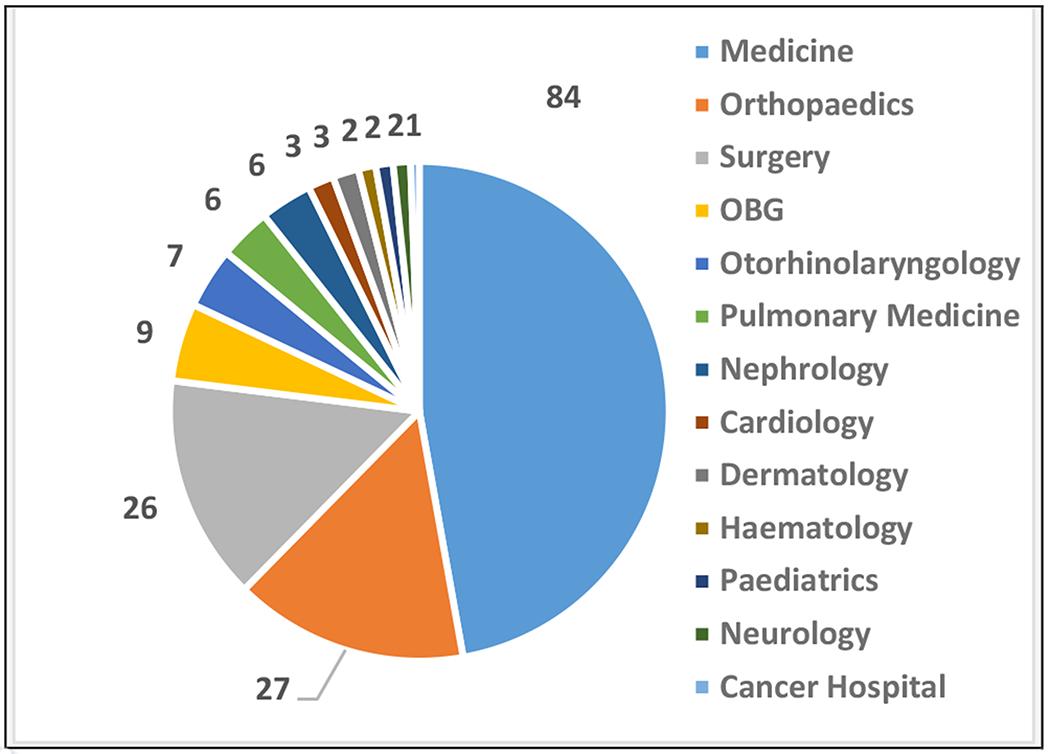
Referring Departments

Mean age of the participants was 36.82 (± 16.96) years. There were 106 men and 72 women. One hundred and forty of the participants were Hindu and the rest 38 were Muslim. Nearly half of the referrals for psychiatric consultation came from the Department of Medicine, followed by departments of Orthopaedics and Surgery (Figure 1). The other departments that asked for psychiatric referral include Obstetrics & Gynaecology, Otorhinolaryngology, Pulmonary Medicine, Nephrology, Cardiology, Dermatology, Haematology, Paediatrics, and Neurology. The Cancer Hospital also gave psychiatric referral.

**Figure 2.**
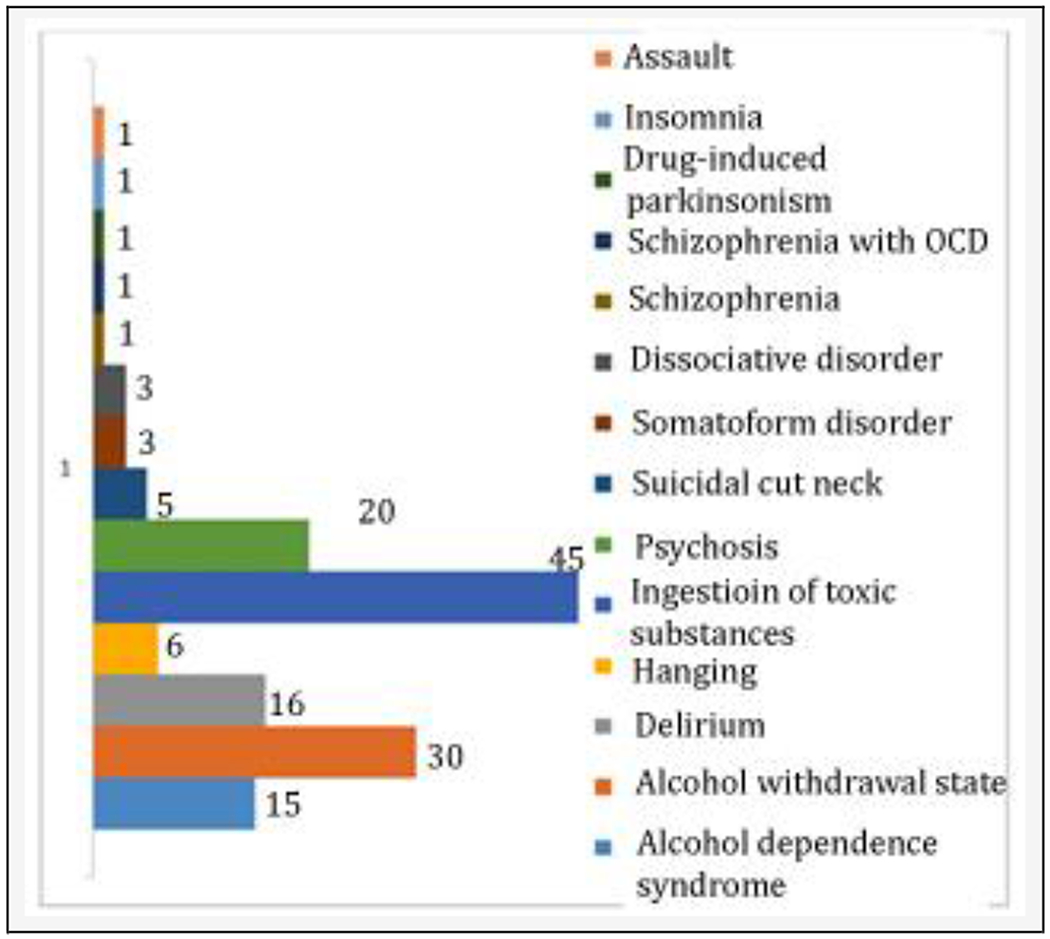
Reasons for Consultation - Obsessive-Compulsive Disorder

Deliberate self-harm and alcohol-related disorders were the two commonest reasons for psychiatric consultation (figure 2). Deliberate self-harms included ingestion of toxic substances, hanging, and cut neck. Alcohol dependence syndrome and alcohol withdrawal state were the alcohol-related disorders. Delirium, psychosis, somatoform disorder, dissociative disorder, schizophrenia, schizophrenia with obsessive-compulsive disorder (OCD), drug-induced Parkinsonism, insomnia, and assault were some other reasons for psychiatric consultation.

**Figure 3.**
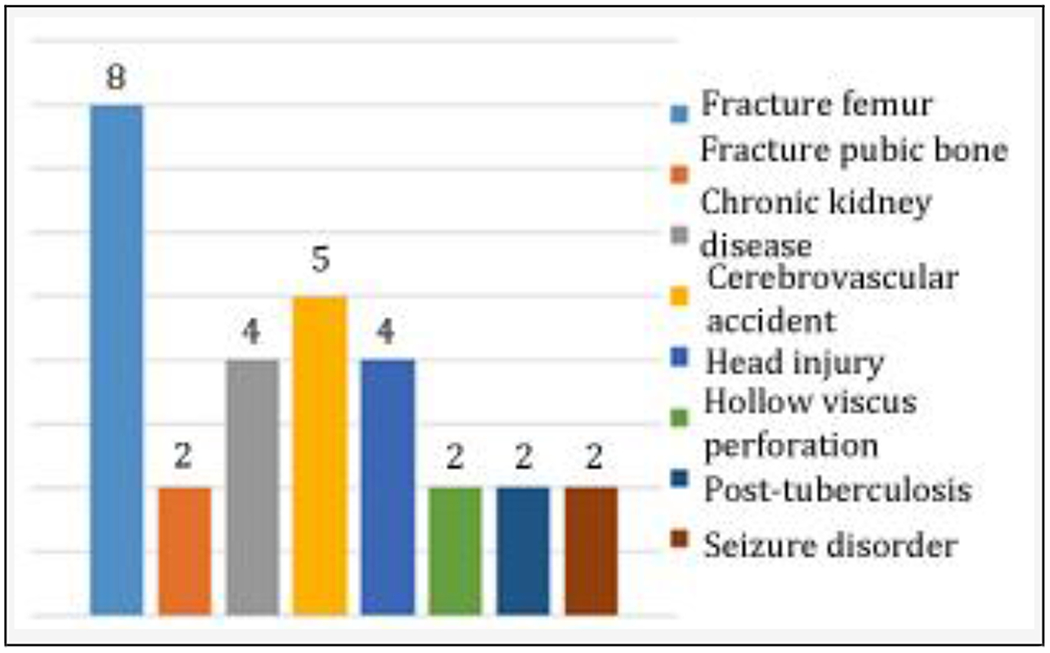
Primary Medical / Surgical Illnesses

Fractures, neurological conditions, injury, acute and chronic diseases were some of the primary medical/surgical illnesses for which the participants were admitted under their respective parent departments (figure 3). The specific medical/surgical illnesses are fracture femur, fracture pubic bone, chronic kidney disease, cerebrovascular accident, head injury, hollow viscus perforation, post-tuberculosis, and seizure disorder. There were follow-up consultations in 22 participants. Most (17) had one follow-up and four had two follow-ups while one was followed up five times. The later, a 50-year-old man was admitted in the Department of Surgery with head injury. He additionally had alcohol withdrawal state.

## DISCUSSION

Our study on consultation-liaison psychiatry in a general hospital from the North-Eastern part of India evaluated a total of 178 participants to find out the referring departments, the reasons for consultation, and the primary medical/surgical illnesses along with their demographic characteristics.

**Figure 4.**
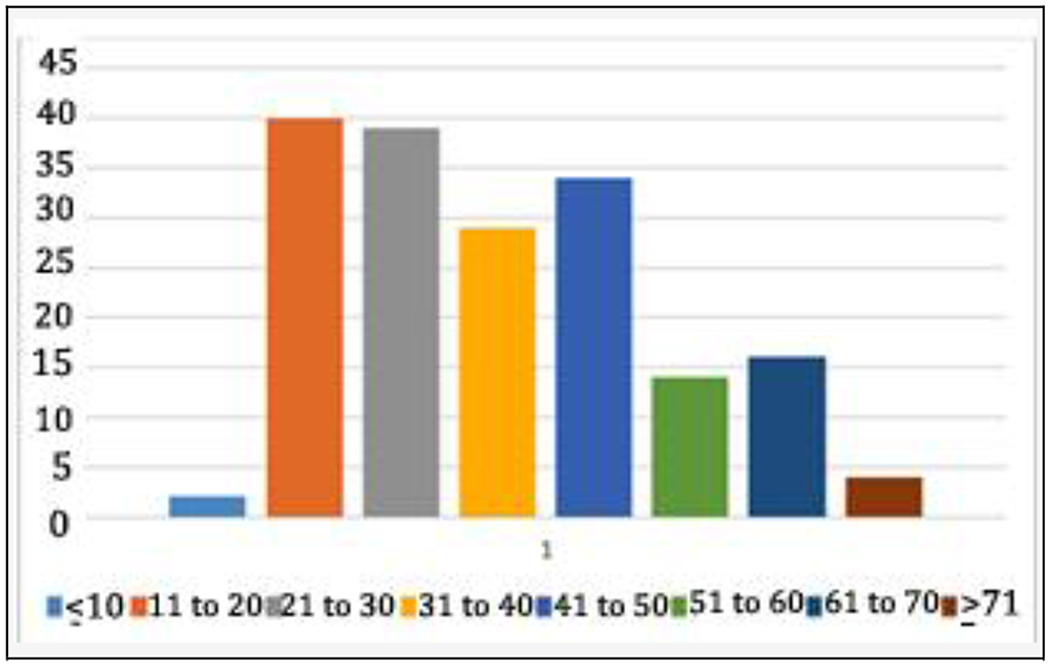
Age Groups in Years

In our study, most of the sample was man (59.6%) (Men: women: 106:72) which is similar to the study by Keertish et al.^[6]^ in which males were 58% and female 42%. The age group was mostly between 11 to 30 years (44.38%) (figure 4] and Hindus outnumbered Muslims (Hindu: Muslim: 140:38).

Majority of the consultations were from the Department of Medicine (47.19%), followed by Orthopaedics (15.17%) and Surgery (14.61%). Singh et al.^[7]^ found that maximum referrals were from Department of Medicine (49.8%) followed by Surgery (11.2%).

Deliberate self-harm was the most common reason for psychiatric liaison (56), that constituted a high number with poisoning (45 i.e. 80.3%) as well as hanging (six) and cut neck (five). Christodoulou et al.^[8]^ found that suicide attempts (146) represent 49.6% of consultations of which 103 (70.5%) were related to self-poisoning. In our study, psychiatric consultation was asked for use of alcohol in a substantial number of patients (45).

It is worth noting here that an earlier work from the same institute studied 748 referrals during a six-month period.^[4]^ Mean age of the patients was about 35 years. Almost 60% were men. Referrals from General Medicine constituted around half of all. General Surgery, Obstetrics and Gynaecology, Otorhinolaryngology, Orthopaedics, Dermatology, Neurology, Endocrinology, and Cardiology were the other referring departments along with the Intensive Care Unit (ICU). Deliberate self-harm and substance use disorder were the most common reasons for referral.

Another work from this part of the globe,^[5]^ looked into the psychiatric consultations in ‘out-of-hours’ casualty/emergency department. Nearly 60% patients were 20-40 years old. Almost half of the consultations were for conversion disorder. This was followed by schizophrenia in about one-quarter. Comorbid medical illness was found in as high as 46%.

Comorbidity of medical/surgical illness with psychiatric disorder is common. This study looked into the psychiatric morbidity in patients with primary medical/surgical illness. Another way to approach the subject is to find out physical/surgical illness in patients admitted to the psychiatry department.

Udey and Niranjan^[9]^ studied physical illnesses in psychiatric inpatients. Physical illnesses were found in 70% patients. These included metabolic, endocrinal, haematological, gastrointestinal, cardiovascular, neurological, and stomatognathic disorders.

Six years’ data of admitted patients in psychiatry department was evaluated and was found that 160 patients had ‘organic mental disorders’.^[10]^ Though the American Psychiatric Association’s Diagnostic and Statistical Manual of Mental Disorders (DSM) has discarded the term,^[11]^ the World Health Organization’s International Statistical Classification of Diseases and Related Health Problems (ICD) still retains it.^[12]^ Seizure disorder was the most common organic mental disorder.

Consultation-liaison psychiatry is also popular as liaison psychiatry or consultative psychiatry. The relation between medical and psychiatric disorder is studied in this field. Practice and teaching are the other two wings of it. Psychiatrists perform it in general hospital. Its services cover diagnosis, therapy, and research. It is like a bridge from psychiatry to the rest of the specialties. Without separating mind and body, this medical practice approaches the individual as a whole being. It reminds us of the psychological aspect of a patient in the doctor-patient relationship. Thus, it has the capacity to improve the quality of life of our patients.^[13]^

The term, ‘liaison psychiatry’ was first coined by Edward Billings. Albany Hospital in New York first provided such service in 1902. The Academy of Psychosomatic Medicine (APM) is an organisation working in this field. Patients admitted in general hospital receive psychiatric consultations. These consultations are more in medical college hospitals.^[13]^ The setting of the present study, i.e. GMCH is such a medical college hospital.

The need for consultation-liaison psychiatry is highlighted by the following facts: “About 500 million people worldwide are believed to suffer from neurotic, stress-related and somatoform disorders. A further 200 million suffer from mood disorders such as chronic and manic depression. Mental retardation affects about 83 million people, epilepsy 30 million, dementia 22 million, and schizophrenia 16 million (WHO, 1999). Surveys of mental morbidity carried out in various parts of India suggest a morbidity rate of not less than 18-20 per 1000 and the types of illness and their prevalence are very much the same as in the other parts of the world.”^[13]^

In more than 40% chronically ill patients, mental disorder sets in during the life lifetime. Substance use disorder and anxiety disorders top this list. Depression and anxiety is often found in chronic illness and pain. The result is functional impairment. There is robust evidence to show the increase in pro-inflammatory cytokines. Immunity goes down. Half of all cancer patients receive psychiatric diagnosis. Risk of depression in diabetes is double compared to non-diabetics. Like in psycho-oncology, the role of consultation-liaison psychiatry is immense in acquired immune deficiency syndrome (AIDS). It has implications not only in prevention and diagnosis but also in treatment and self-responsibility. Good prognosis and reduced cost are achieved when mental health is incorporated in general healthcare.^[13]^

To sum up, liaison psychiatry deals with detection and treatment of mental and behavioural disorders in people with general medical conditions. There is a particular uniqueness to this sub-specialty of psychiatry. It does not address specific disorders. It also does not involve certain age groups. Rather, certain clinical settings determine its uniqueness. Division of the psychiatrists from colleagues of other medical and surgical specialties of medical sciences led to this development. For example, there is no liaison surgeons. A surgeon will walk into a medical ward and do the needful as routine. This development is relatively new. One of the major reasons is the lack of outside referral for psychiatric care. But, high occurrence of mental and behavioural disorders in the patients with medical and surgical illnesses is beyond doubt. Moreover, specialisation of medical services results in need for experts attending to disorders of their disciplines. Concerned hospitals delineate the role to be played by the liaison psychiatrists.^[14]^

The liaison psychiatrist needs to diagnose new mental disorder in a patient having a general medical illness. If the patient with general medical illness also has mental disorder from before, that too needs management by the liaison psychiatrist. Many patients with mental disorders have somatic presentations. Likewise, medical conditions too have associated psychological problems. Both are to be addressed by the liaison psychiatrist. Often, medically unexplained conditions are encountered. Similarly, there can be different behavioural disturbances as well. These also call the liaison psychiatrist into the picture. Suicide and deliberate self-harm, alcohol and drug abuse, childbirth and puerperium, capacity and legal powers are some additional frontiers to work upon by the liaison psychiatrist.^[14]^

Liaison psychiatry is also referred to as consultation-liaison psychiatry. The latter is a better term to describe the different approaches. Sometimes the psychiatrist of this subspecialty needs to work hand in hand with the other specialties. At other times the psychiatrist of this subspecialty may need to take over the care of the patient. There do exist some differences as far as the presentation of patients are concerned if compared between those in consultation-liaison psychiatry and those in the general population. This list can be as follows: “adjustment disorder, depressive illness, patients presenting after parasuicide, alcohol problems, drug problems, acute confusional state, psychiatric aspects of organic conditions, medically unexplained symptoms, behavioural disturbance, assessment of dementia, postpartum illnesses, illnesses specific to women, questions regarding capacity, issues of consent to/refusal of treatment”.^[14]^

The workplace of a liaison psychiatrist is the general hospital. This is a situation for bouquets and brickbats. The various specialists running the general hospital have their own concept of psychiatry and their way of approaching patients. These may not always be the same as practiced by a psychiatrist. On the other hand, they may lack the skills that a psychiatrist has in the armoury. Therefore, a controlled balance can go a big way in enriching the endeavour of catering to the need of mental and behavioural health in patients with general medical condition.^[14]^

The working environment may not give at home feeling initially. Many medical conditions are expected to be encountered about which the liaison psychiatrist will know little. Therefore, it is important to study and record the atmosphere closely. The liaison psychiatrist has to try to know why the colleague from the other medical and surgical specialties have asked for psychiatric consultation. Patients should understand that psychiatric referrals are sought.^[14]^

Reviewing earlier records is rewarding. Discussion with colleagues from other specialties always helps. Seniors of the treating team can be a guiding force. Clarification of doubts is enriching. Nurses’ inputs give a 24 x 7 scenario of the patients. If possible, privacy for interview with patients is desired. Introduction should be as psychiatrist or psychological medicine specialist. This follows explanation about why the consultation is made. If exists, prompt clarification has to be made to remove any “Am I crazy”-kind of thought. Clear notes are to be made. Plan has to be penned down. Recommendation for management is made. Need for review is documented. Face to face clarification of points with the other treating team members is preferred.^[14]^

Assam, with a population of 31,169,272,^[15]^ is one of the eight North-Eastern states of India. India is a low- and middle-income country (LAMIC).^[16,17]^ An interesting piece of work looked into the indexation of psychiatric journals in major bibliographic databases.^[18]^ What was found is that most of these journals are from high income countries. This gap is so huge that it is called the ‘5/95’ gap.^[19]^ That is, on an average of 100 psychiatric journals getting indexed in major bibliographic databases, only five are from LAMIC. Eighty per cent of the world population lives in LAMIC. Obviously, most of the burden from mental and behavioural disorders, including substance use disorders are borne by population from these regions. Yet, our policy making on health in general and psychiatry in particular is mostly North America or Western Euro-centric. In other words, 20% of the word population decides what is best for the rest 80%. Thus, it is pertinent that scientific literature has a global representation. Work of the kind published in the current study fills that gap. There are certain publications working on reducing this chasm between high income countries and LAMIC by the shared knowledge of brain and behavioural sciences. The Open Journal of Psychiatry & Allied Sciences (OJPAS®), formerly Dysphrenia is one such endeavour.^[20]^

We need not stand still on whatever laurels that has been achieved this far. Instead we need to roll on. We need to enrich ourselves more. Our journey has made us know what we know. In the meantime, we have also known what we do not know. Though, we need to remain humble. There is a bigger fact in front of us. That fact is there are things “we don’t know we don’t know”!^[21]^

### Limitations

Retrospective chart-review studies limit from analysing many other variables intended to look into along with the inherent bias of missing data. Short study period limits from studying the pattern of consultations throughout the year.

### Strengths

GMCH is a tertiary care teaching hospital. Data from the leading and premier general hospital from the North-Eastern part of India throws light on the subject of consultation-liaison psychiatry and thus, enriches the scientific literature base from this region.

### Implications

Medical/surgical comorbidity are high in the population with psychiatric disorders and the vice versa. This study helps in making the psychiatrists working in a general hospital setting to equip oneself in this field. Moreover, physicians and surgeons from the other departments of a general hospital also become aware for the need of addressing the psychiatric comorbidity from this type of study.

### Future Directions

Extension of the study over a longer period will help in knowing is there any change in pattern of psychiatric consultations over time.

## CONCLUSIONS

Maximum psychiatric consultation was sought from the Department of Medicine and the commonest reason was for deliberate self-harm. Attending to psychiatric comorbidity along with treatment for the surgical/medical illnesses gives a holistic approach towards our patients that favourably influences the course of illness and also improves the quality of life.

## Figures and Tables

**Figure 5.**
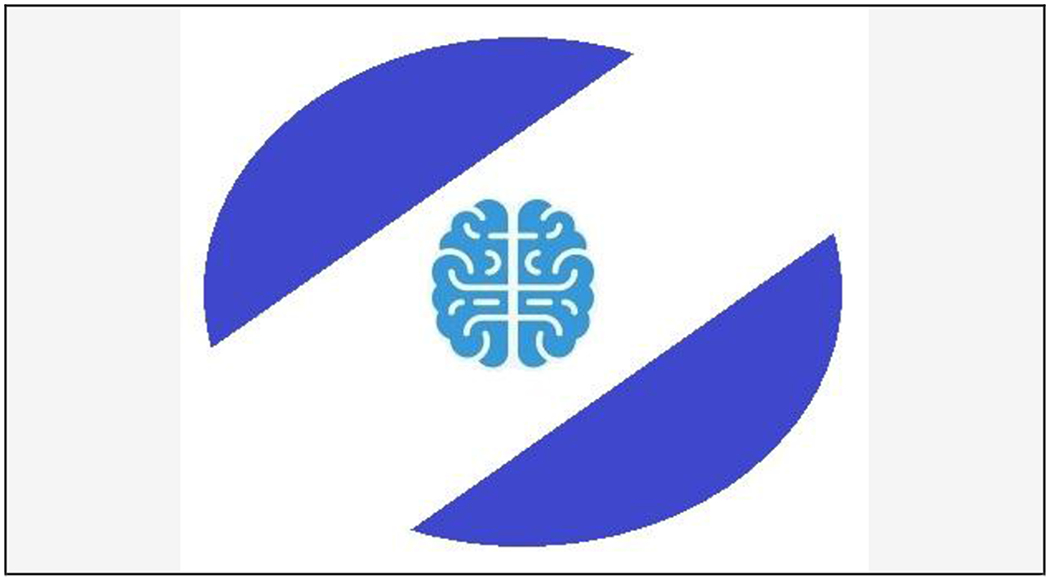
Global Psychiatry- A LAMIC Perspective. LAMIC= Low- and Middle-Income Countries
